# The succinate prodrug NV354 prevents brain lesions and late-stage motor dysfunction in mitochondrial complex I deficiency

**DOI:** 10.1016/j.isci.2026.114717

**Published:** 2026-01-16

**Authors:** Meagan J. McManus, Yi Zhu, Cesar Alves, Neha Kohli, Patricia Prada-Dacasa, Laura Sanchez-Benito, Elisenda Sanz, Irene Yee, Lozen Robinson, Malkah Sheldon, Walter J. McHugh, Abhay Ranganathan, Jennie Meng, Nina Duncan, Alvar Grönberg, Douglas C. Wallace, Sarah Piel, Michael Karlsson, Steven J. Moss, Lee Webster, Magnus J. Hansson, Eskil Elmér, Johannes K. Ehinger, Albert Quintana, Todd J. Kilbaugh

**Affiliations:** 1 Resuscitation Science Center of Emphasis, The Children’s Hospital of Philadelphia Research Institute, Philadelphia, PA, USA; 2 Anesthesiology and Critical Care Medicine, The Children’s Hospital of Philadelphia, Philadelphia, PA, USA; 3 Center for Mitochondrial and Epigenomic Medicine, The Children’s Hospital of Philadelphia, Philadelphia, PA, USA; 4 Center for Innovation in Brain Science, University of Arizona Health Sciences, Tucson, AZ, USA; 5 Department of Pharmacology, College of Medicine Tucson, University of Arizona, Tucson, AZ, USA; 6 Division of Neuroradiology, Department of Radiology, The Children’s Hospital of Philadelphia, Philadelphia, PA, USA; 7 Mitochondrial Neuropathology Laboratory, Institut de Neurociències and Department of Cell Biology, Physiology and Immunology, Universitat Autònoma de Barcelona, Bellaterra, Spain; 8 Mitochondrial Medicine, Department of Clinical Sciences Lund, Lund University, BMC A13, Lund 221 84, Sweden; 9 Abliva AB, Medicon Village, Lund 223 81, Sweden; 10 Department of Neurosurgery, Rigshospitalet, Copenhagen, Denmark; 11 Isomerase Therapeutics Ltd, Chesterford Research Park, Cambridge CB10 1 XL, UK; 12 Otorhinolaryngology, Head and Neck Surgery, Department of Clinical Sciences Lund, Lund University, Skåne University Hospital, Lund, Sweden

**Keywords:** natural sciences, biological sciences, biochemistry, neuroscience

## Abstract

Leigh syndrome is a fatal pediatric neurodegenerative disease caused by mitochondrial dysfunction, which can be modeled in the *Ndufs4* KO mouse with mitochondrial respiratory chain complex I (CI) deficiency. This study explores NV354, a prodrug of succinate with enhanced oral bioavailability and brain uptake, as a potential therapy to counteract this devastating condition. NV354 modulated whole-body respiration and metabolic flexibility, prevented late-stage motor dysfunction, delayed clinical ataxia scores, and improved body weight development, but had otherwise minimal effect on neurobehavior and lifespan of the animals. The succinate prodrug prevented development of the brain stem lesions pathognomonic for Leigh syndrome, attenuated neuronal loss in the brainstem, diminished activation of astrocytes, blocked hypertrophic microglial accumulation, and reduced reactive oxygen species (ROS) levels in the brain. NV354 also partially alleviated motor symptoms and metabolic decompensation in a rat model of Parkinson disease induced by the CI inhibitor rotenone. In conclusion, the succinate prodrug NV354 shows promise as a potential treatment of mitochondrial CI-related neurodegeneration.

## Highlights

• Succinate prodrug prevents brain lesions and motor dysfunction in mitochondrial disorders
• Succinate prodrug reduces oxidative stress and neuroinflammation in mice
• Therapeutic effects observed in complex I deficiency and Parkinson disease models
• Succinate prodrugs may provide a novel treatment for mitochondrial disorders

## Graphical abstract

**Figure:**
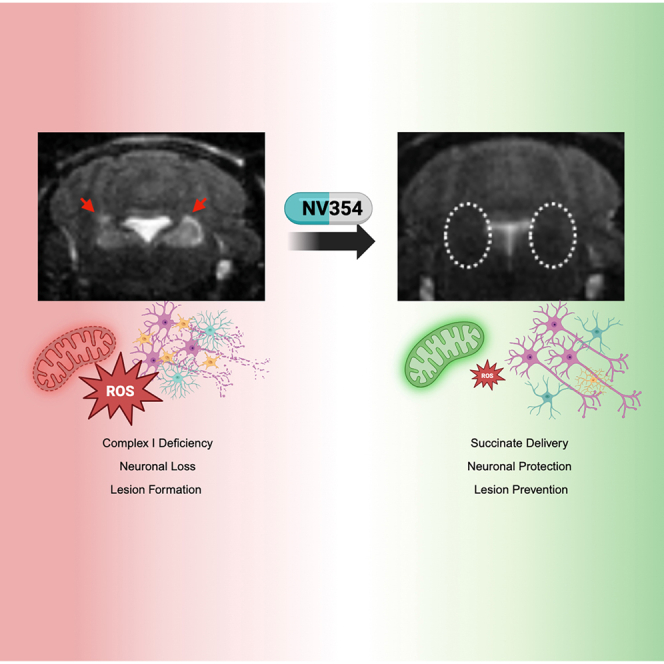

## Introduction

Mitochondrial diseases are a clinically heterogeneous group of disorders arising from dysfunction of the mitochondria, the primary energy-producing organelles in eukaryotic cells. These diseases are often multisystemic, affecting tissues and organs with high energy demands such as the brain, heart, and muscles. Neurological impairment is a common manifestation, reflecting the brain’s critical dependence on mitochondrial bioenergetics. Primary mitochondrial diseases (PMDs) can result from mutations in either nuclear DNA (nDNA) or mitochondrial DNA (mtDNA), affecting a wide range of mitochondrial processes, including oxidative phosphorylation (OXPHOS), mitochondrial dynamics, and metabolite transport. Complex I (CI), or NADH:ubiquinone oxidoreductase, is the first and largest enzyme complex in the electron transport chain (ETC). CI oxidizes NADH, using the energy to pump protons across the inner membrane and generate the proton-motive force necessary to drive ATP synthesis. CI is also a major site of reactive oxygen species (ROS) production. These critical roles in metabolism and cell signaling make CI dysfunction one of the most common causes of PMDs.

Impairment of CI function has been implicated in a spectrum of neurological disorders, ranging in severity from relatively mild to severe, early-onset encephalopathies. At the severe end of this spectrum lies Leigh syndrome (LS),^1^ a devastating pediatric neurodegenerative disorder characterized by progressive motor and cognitive decline, respiratory abnormalities, and often early mortality. Mutations in over 75 genes have been associated with LS, highlighting its extensive genetic heterogeneity, and mutations in CI subunits account for a large percentage of cases.^2^^,^^3^ Beyond primary genetic defects, impaired CI function has also been linked to Parkinson disease (PD),^4^ the second most common neurodegenerative disease worldwide. PD is clinically characterized by the progressive loss of dopaminergic neurons in the substantia nigra, leading to motor dysfunction and non-motor symptoms. While the etiological significance of mitochondrial CI genetics in PD remains uncertain,^5^ toxin-induced CI inhibition using agents like rotenone is known to recapitulate features of PD in animal models,^6^^,^^7^ providing a valuable platform for studying potential therapeutic interventions.

One of the most commonly affected genes in LS is *NDUFS4* (nuclear-encoded NADH:ubiquinone oxidoreductase core subunit S4), which encodes an 18 kDa supernumerary subunit critical for CI assembly and stability.^8^ NDUFS4 interacts with both the N-module (the NADH-dehydrogenase module) and the Q-module (ubiquinone-reducing module) in CI. The absence of NDUFS4 disrupts the stable attachment of the N-module to the rest of the complex, impacting CI assembly and activity.^9^ This crucial role of NDUFS4 in CI directly explains the deleterious effects of *NDUFS*4 mutations in LS. Patients harboring pathogenic mutations in *NDUFS4* develop lesions in the basal ganglia and brainstem, and these lesions are observed on magnetic resonance imaging (MRI) with T2-weighted imaging (T2WI) as hyperintense regions that are considered pathognomonic for LS. These lesions are also frequently associated with breathing abnormalities, seizures, ataxia, and dystonia.^1^^,^^10^^,^^11^^,^^12^^,^^13^ Loss of murine *Ndufs4* recapitulates many salient features of LS, including these bilateral lesions, as well as growth regression, glial activation, seizures, ataxia, breathing abnormalities, and premature death.^14^^,^^15^^,^^16^

In CI deficiency, NADH metabolism is restricted, the function of complex II (CII) is upregulated, and cellular succinate levels are decreased.^17^^,^^18^^,^^19^ We hypothesized that facilitated intracellular delivery using a prodrug strategy may restore succinate supply to critical metabolic reactions, such as the tricarboxylic acid (TCA) cycle and other signaling pathways. We have previously shown that cell-permeable succinate prodrugs deliver succinate to the mitochondria, restoring OXPHOS in cells from LS patients.^20^ However, the first-generation succinate prodrugs were not suitable for *in vivo* testing due to low plasma stability. Here, we present a second-generation succinate prodrug with improved plasma stability and oral bioavailability that enters target organs and displays neuroprotective properties in animal models of mitochondrial CI dysfunction.

## Results

### Pharmacokinetics and toxicity assessment of the succinate prodrug NV354

**Figure 1:**
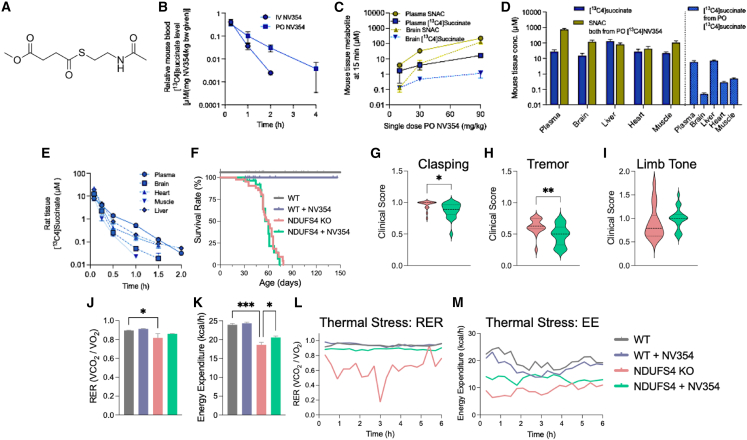
Pharmacokinetic profiling and therapeutic potential of the succinate prodrug NV354 in *Ndufs4* KO mice (A) Structure of NV354. (B–D) Pharmacokinetic profiling in CD-1 mice (*n* = 3 per time point). Mean blood concentration-time profiles of [13C]succinate after single intravenous (i.v.) or oral gavage (PO) administration of [13C]-NV354 (B). NV354 metabolite concentrations in the plasma and brain 15 min following single PO dose of 10, 30, and 90 mg/kg (C). Distribution of NV354 metabolites ([13C]NV354:succinate and SNAC) or equimolar [13C4]-succinate 15 min after administration via PO (D). (E) Concentration of [13C4]-succinate metabolite over time following [13C]-NV354 i.v. administration (20 mg/kg) in rats. (F) Kaplan-Meier curves showing no change in mean survival of *Ndufs4* KO mice (60.3 days) with NV354 provided via drinking water (DW, 250 mg/kg/day) by Mantel-Cox log-rank test, *n* = 14–59. (G–I) Ataxia sickness scores measured in *Ndufs4 KO* mice by trunk curl/hindlimb clasping (G) and intermittent tremor (H) were decreased by NV354 during late-stage disease (≥45 days), but paralysis of either hindlimb was unaffected (I), *n* = 14–59, unpaired *t* test. (J and K) Reduced mean respiratory exchange ratio (J; RER) and energy expenditure (K; EE) were partially restored by NV354 treatment in 30 days old mice during the dark cycle (19:00-7:00) at RT (∗*p* < 0.01; ∗∗*p* < 0.001). (L and M) NV354 prevented metabolic decompensation in *Ndufs4* KO during thermal stress (6 h, 30°C), as measured by RER (L; *p* = 0.015 for *Ndufs4* KO vs. WT; *p* = 0.81 for *Ndufs4* KO + NV354 vs. WT) and partially restored energy expenditure (M; *p =* 0.0009 for *Ndufs4* KO vs. WT; *p* = 0.03 for *Ndufs4* KO + NV354 vs. WT) by two-way ANOVA followed by Holm-Šídák’s posthoc test for multiple comparisons. Bar graph values represent the mean ± SEM; *n* = 6–8. See also Tables S1 and S2 and Figures S1–S4.

NV354 is a stable, water-soluble prodrug of succinate (Figure 1A). It is soluble at >190 mg/mL in PBS (pH 7.4) or tap water, with no degradation observed when stored at 4 °C for 11 days. A PK profile of NV354 was generated using intravenous (i.v.) and per oral (PO, as oral gavage) administration of [^13^C_4_]NV354 in CD1 mice (Figures 1A–1D). Orally (PO) administered [^13^C_4_]NV354 resulted in slower elimination of [^13^C_4_]succinate compared with i.v. administration (Figure 1B). Next, we assessed the oral bioavailability of NV354. Because NV354 is metabolized as it releases succinate, the parent compound is in flux and an absolute %F is not meaningful; bioavailability was therefore inferred from levels of NV354 metabolites, released [13C4]succinate, and the cleaved side‑chain N-acetylcysteamine (SNAC) in plasma and brain. Labeled succinate and SNAC increased with the increasing NV354 dose (10–90 mg/kg) in the brain and plasma. The [^13^C_4_]succinate increase was approximately dose proportional (3-fold per step) in plasma and brain. In contrast, the SNAC increase was greater than the dose proportional increase in plasma (6-fold per step) and brain (30-fold per step) (Figure 1C). Fifteen minutes following PO administration, NV354 delivered at least 1 and up to >2 orders of magnitude (to the brain) more [^13^C_4_]succinate to the target organs compared with the equimolar administration of [^13^C_4_]succinate. The distribution of the other NV354 metabolite, the SNAC side-chain, was highest in the brain (Figure 1D). [^13^C_4_]NV354 i.v. administration to Sprague-Dawley rats at 20 mg/kg likewise resulted in [^13^C_4_]succinate delivery to the tested target tissues (Figure 1E). Plasma half-life (*t*_*1/2*_*)* of NV354 was assessed by incubation of NV354 in mouse, rat, and human plasma, and NV354 was quantified at serial time points using mass spectrometry (t_1/2_ [min]: human, 52.2; rat, 1.12; mouse, 0.887). In summary, despite the very short half-life in rodent plasma as compared to human plasma, the distribution of NV354 metabolites following PO administration confirms that its plasma stability and bioavailability are sufficient for organ uptake, with highest uptake in the brain, and that the succinate delivered is metabolized in target organs.

Toxicity screens for chronic NV354 oral administration were performed in C57B6/J mice (Figure S1). NV354 (50–750 mg/kg) was administered via daily PO starting at wean (postnatal day 19) for up to 60 days (*n* = 15–19). NV354 was well tolerated, with toxic effects seen only at the highest dose (750 mg/kg), which reduced median lifespan to 76 days (Figure S1A; *p* = 0.011 vs. baseline; Mantel-Cox log rank test) and also reduced weight after 10 days of treatment (Figure S1B; *p* < 0.001 vs. baseline), but had no effect on surface body temperature (Figure S1C; *p* = 0.60). The highest dose of NV354 also led to contracted body contour indicative of anxiety or pain (Figure S1D; *p* < 0.05 vs. baseline). There were no changes in motor function of C57B6/J mice as measured in the open field test (Figure S1E)*.* At 250 mg/kg, NV354 increased the total distance traveled in running wheels and increased energy expenditure measured in metabolic cages (Figure S1F and S1G, *p* < 0.05 vs. baseline). Since long-term daily PO treatment can be stressful for the animals, and our PK results suggested NV354 is quickly metabolized, we next tested the tolerability of NV354 in the drinking water. In the oral dosing screen, NV354 appeared well tolerated, as there were no changes in mouse weight or water consumption with NV354 concentrations up to 700 mg/kg in the drinking water, measured over 5 days (Figure S2). Taken together, these results suggested that chronic administration of 250 mg/kg NV354 via the drinking water may be the optimal tolerable delivery regimen of the succinate prodrug to mice with complex I impairment.

### Therapeutic efficacy and metabolic impact of the succinate prodrug in *Ndufs4* KO mice

To determine the therapeutic efficacy of NV354 in primary mitochondrial disease, *Ndufs4* KO mice were provided ad lib access to 250 mg/kg NV354 in the drinking water from the time of weaning to mortality (henceforth termed *Ndufs4* KO + NV354). Analysis of NV354’s SNAC sidechain revealed higher levels in *Ndufs4* KO plasma but equimolar concentration in the brain of WT and *Ndufs4* KO mice treated with NV354 for approximately 60 days (Figure S3). Oral NV354 had no effect on *Ndufs4* KO median lifespan (Figure 1F) or body weight (*p* = 0.33; *n* = 49–52). Surface temperature was decreased 0.5 ± 0.12°C in *Ndufs4* KO relative to WT mice (*p* = 0.0117), which was normalized by NV354 treatment (*p* = 0.877 for *Ndufs4* KO vs. WT). Conversely, oral NV354 decreased the temperature of WT mice by 0.43 ± 0.06°C (*p* < 0.0001 for WT vs. WT + NV354). Next, motor function was assessed in *Ndufs4* KO mice at the mid-stage (30 days) of complex I disease using the open field test. *Ndufs4* KO mice were impaired in every measure of motor function (Figures S4A–S4E; *p* < 0.001 relative to WT), and oral NV354 treatment had no effect. Motor function of *Ndufs4* KO mice progressively declines with age, and the mice show signs of ataxia that can become debilitating by ∼45 days of age. To determine if NV354 could prevent further motor deterioration, evidence of ataxia was quantified daily from 45 days of age to end of life. Oral NV354 prevented late-stage motor dysfunction in *Ndufs4* KO mice as measured by trunk curl/hindlimb clasping, paralysis of one or both hind limbs, and intermittent tremor (Figures 1G–1I; *p <* 0.01 for *Ndufs4* KO vs. *Ndufs4* KO + NV354).

Next, the effect of NV354 on *Ndufs4* KO whole-body respiration and metabolic flexibility was determined by monitoring 30 days old mice in calorimetry cages. The results show that loss of *Ndufs4* reduces the respiratory exchange ratio (RER; VO_2_/VCO_2_), suggesting that *Ndufs4* KO mice shift to lipid oxidation (Figure 1J; *p* < 0.05), perhaps to enhance thermogenesis.^21^ NV354 treatment resulted in a trend toward normalization of *Ndufs4* KO RER (*p* = 0.585 for *Ndufs4* KO + NV354 vs. WT). This trend, together with the improved energy expenditure (kcal/h) in *Ndufs4* KO mice observed in Figure 1K (*p* = 0.011 for *Ndufs4* KO vs. *Ndufs4* KO + NV354), suggests a potential improvement in overall energy metabolism in *Ndufs4* KO mice treated with NV354. Neither metabolic parameter can be attributed to changes in activity as NV354 had no effect on total movement of *Ndufs4* KO mice in the home cage environment (Figure S4H).

Dysregulation of body temperature homeostasis and sensitivity to thermal stress are commonly observed in LS patients and *Ndufs4* KO mice.^15^^,^^22^^,^^23^ To determine if NV354 could prevent metabolic decompensation in *Ndufs4* KO caused by thermal stress, murine metabolism was measured during exposure to 6 h of heat stress at 30°C. The RER declined significantly at midpoint of the temperature stress (3 h) in *Ndufs4* KO mice, but NV354 maintained the RER of *Ndufs4* KO mice to the level of WT mice (Figure 1L; F^3^^,^^24^ = 4.103, *p* = 0.0175; WT = 0.94 ± 0.1, *Ndufs4* KO = 0.63 ± 0.1, *Ndufs4* KO + NV354 = 0.88 ± 0.1) and partially restored energy expenditure (Figure 1M; F^3^^,^^24^ = 7.024, *p* = 0.0015; WT = 19.9 ± 2.4, *Ndufs4* KO = 9.2 ± 2.5, *Ndufs4* KO + NV354 = 13.3 ± 2.4), suggesting that NV354 increased resilience to stress.

### Neuroprotective effects of the succinate prodrug on brainstem lesions and neuroinflammation in *Ndufs4* KO mice

**Figure 2:**
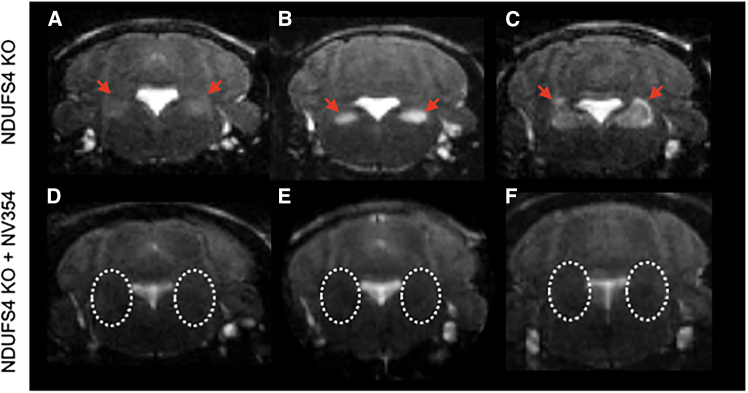
NV354 prevents the emergence of bilateral, hyperintense lesions in the brainstem of *Ndufs4* KO mice (A–C) Representative axial head T2 MRIs showing mild (A), moderate (B), and severe (C) symmetrical hyperintense lesions in the vestibular nuclei (red arrows) of *Ndufs4* KO mice over 45 days of age. (D–F) Representative axial head T2 MRIs showing the absence of bilateral, hyperintense lesions (dashed circles), as well as reduced size of the fourth ventricle in age-matched NV354-treated mice (DW, 250 mg/kg/day) (*p* = 0.003) by unpaired *t* test, *n* = 7–11.

Despite the heterogeneous clinical presentation of LS, bilateral hyperintense lesions within the brainstem of patients are a unifying feature of the disease.^3^^,^^24^ Using T2-weighted MRI, hyperintense, bilateral lesions were detected within the vestibular nuclei (VN) of the brainstem of *Ndufs4* KO mice over 45 days of age (Figures 2A–2C). In untreated mice, these lesions varied in severity and were classified based on consideration of signal intensity, homogeneity, and size. Homogeneous lesions (Figures 2A and 2B) are indicative of predominant edema and may reverse in mitochondrial disease patients,^24^ whereas the more severe heterogeneous lesions (Figure 2C) reflect potential areas of necrosis, liquefaction, or hemorrhage. Faint, homogeneous lesions were quantified as mild (Figure 2A), bright homogenous lesions as moderate (Figure 2B), and heterogeneous lesions with the presence or absence of tumefactive appearance (lesions extending beyond the anatomic limits of the primary region affected) were quantified as severe (Figure 2C).

Seventy-five percent of untreated *Ndufs4* KO mice presented with VN lesions, and 33% of these lesions were moderate to severe (*n* = 8). Conversely, only one of eleven (9%) age-matched, NV354-treated *Ndufs4* KO presented with VN lesions (Figures 2D–2F; *p* = 0.0025, unpaired *t* test).

**Figure 3:**
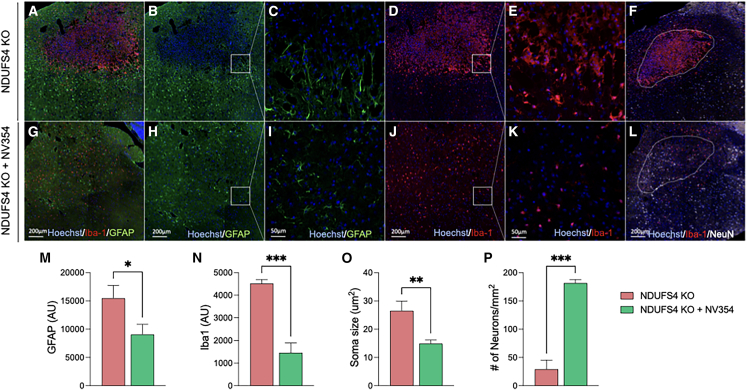
NV354 attenuates late-stage gliosis and loss of neurons in the VN of *Ndufs4* KO mice (A–F) Representative immunofluorescence staining images for nuclei (Hoechst; blue), microglia (Iba1; red), astrocytes (GFAP; green), and neurons (white) in the VN of *Ndufs4* KO mice (untreated) compared to *Ndufs4* KO + NV354 (DW, 250 mg/kg/day) at approximately 60 days of age (G–L). (A–C) Activated astrocytes (green) were present on the periphery, but largely excluded from the lesion site (C) higher-magnification of the inset in (B), highlighting the lesion border. (D–F) Hypertrophic microglia (red) localized to the VN lesion of *Ndufs4* KO mice. (G–L) NV354 treatment diminished activation of astrocytes (H–I) and hypertrophic microglial localization (J–L) in the brainstem of *Ndufs4* KO mice. Loss of neurons within the VN of *Ndufs4* KO (F) was prevented by NV354 treatment (L). (M–P) Quantification of the fluorescence intensity (AU) of GFAP (M) in the VN region, and Iba1 fluorescence intensity (N), mean microglial (Iba1+) soma size (O), and the number of NeuN-stained neurons (P) within the lesion area (F, L white dashed ROI) of *Ndufs4* KO mice, ∗*p <* 0.05; ∗∗*p <* 0.01; ∗∗∗*p <* 0.001 by unpaired *t* test, *n* = 3. Bar graph values represent the mean ± SEM. See also Figure S5. VN, vestibular nuclei.

These LS-related lesions are characterized by increased glial reactivity, neuronal death, and oxidative stress.^14^ To determine the effect of NV354 on neuroinflammation and neuronal viability in the brainstem, we investigated the relationship between NV354 treatment and these characteristics. The results showed hypertrophic microglia (Iba1; red) localized to the site of the lesion in the brainstem of *Ndufs4* KO mice at approximately 60 days of age (Figures 3A and 3D–3F). Astrocytes (GFAP; green) appeared activated and prominent throughout the brainstem and other regions (Figure S5) but were largely excluded from the lesion site (Figures 3B and 3C). NV354 treatment diminished the activation of astrocytes (Figures 3H and 3I) and prevented hypertrophic microglial accumulation (Figures 3J–3L) in the brainstem of *Ndufs4* KO mice. Since NV354 prevented hypertrophic microglial activation, a response that promotes neuronal loss in the *Ndufs4* KO VN,^25^ NeuN-stained neurons (white) were quantified within the lesion site or anatomically matched region (Figures 3F and 3L). The results reveal that NV354 preserved neurons within the VN region of *Ndufs4* KO mice (Figure 3M; *p* = 0.0009, unpaired *t* test).

### Mitigation of oxidative stress by the succinate prodrug in the *Ndufs4* KO brain and human cells with CI impairment

**Figure 4:**
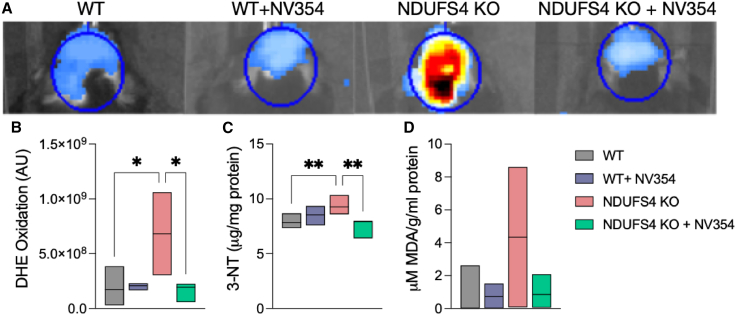
NV354 reduces oxidative stress in the *Ndufs4* KO brain (A) ROS detected *in vivo* by DHE oxidation in the mouse brain at 30 days of age. Representative images are shown. (B) NV354 (DW, 250 mg/kg/day) attenuated DHE oxidation by ROS in *Ndufs4* KO brain (∗*p* < 0.05). (C) NV354 prevented oxidative damages measured by 3-NT (∗∗*p* < 0.01). (D) Lipid peroxidation measured by MDA adducts (*p* = 0.09 for *Ndufs4* KO vs. WT) by one-way ANOVA followed by Šídák’s posthoc test for multiple comparisons, *n* = 3–8. Bar graph values represent the mean ± SEM. ROS, reactive oxygen species; DHE, dihydroethidium; 3-NT, 3-nitrotyrosine; MDA, malondialdehyde.

Since mitochondrial CI impairment leads to oxidative stress, and oxidative stress is associated with glial activation, which occurred at the site of MRI lesions, we next investigated whether NV354 could potentially mitigate these processes by decreasing ROS levels and downstream oxidative damage. Superoxide and associated ROS were detected *in vivo* by DHE oxidation and quantified using the In Vivo Imaging System (IVIS) optical imager (Figure 4A). The results showed a dramatic increase in ROS in the *Ndufs4* KO brain during the early stage of disease (30 days of age). NV354 treatment completely blocked this increase, restoring DHE oxidation levels to that of WT mice (Figure 4B; *p* = 0.9995 for *Ndufs4* KO + NV354 vs. WT). NV354 had no appreciable effect on ROS in WT animals *in vivo* (Figure 4B; *p >* 0.9999 for WT + NV354 vs. WT). To investigate the mechanism of ROS reduction by NV354, we treated human PMBCs with mitochondrial inhibitors and compared the effects of NV354 with the SNAC moiety alone. Following respiratory CI inhibition with rotenone (2 μM), flow cytometric analysis revealed an increased DHE (16%, *p* = 0.03) mean fluorescence intensity (MFI) as compared to non-inhibited samples. The increase in the MFI of MitoSOX Deep Red was similar (15%) but non-significant (data not shown). The addition of NV354 (100 μM) decreased MitoSOX Deep Red and DHE in non-inhibited (Figure S6A) as well as in rotenone-inhibited human peripheral blood mononuclear cells (PBMCs) (Figure S6B, *p* < 0.05; *n* = 7; Wilcoxon matched-pairs signed-ranks test), as compared to cells treated with equimolar concentration of the cleaved byproduct SNAC. Conversely, addition of NV354 to cells with a downstream block on complex III (CIII) of the respiratory chain with antimycin A (AMA; 1 mg/mL) increased both MitoSOX Deep Red and DHE MFI as compared to cells treated with equimolar concentration of SNAC (Figure S6C, *p* < 0.05; *n* = 7). Thus, NV354 decreased ROS in non-inhibited, as well as CI-inhibited cells, but not in cells with CIII inhibition, suggesting that the effect is specifically related to metabolism of the released succinate.

While increased ROS production in cells derived from LS patients has been consistently reported in the literature using a similar assay,^26^^,^^27^ evidence of downstream oxidative damages in *Ndufs4* KO mice is conflicting.^15^^,^^28^^,^^29^^,^^30^ We found increased oxidative damage to proteins, as measured by 3-nitrotyrosine (3-NT) adducts (Figure 4C; *p* < 0.01 for *Ndufs4* KO vs. WT), and a trend toward increased lipid peroxidation (Figure 4D *p* = 0.09 for *Ndufs4* KO vs. WT), as measured by malondialdehyde (MDA), in the late-stage of *Ndufs4* KO disease (∼60 days of age), which were reduced by NV354.

Since NV354 is metabolized rapidly, and there is some variability in dose delivery via ad lib drinking water, we also performed a series of experiments with controlled delivery of NV354 via osmotic pumps. The subcutaneous osmotic mini pumps were implanted at day P23, with continuous infusion of NV354 (70 mg/kg/day) or vehicle to *Ndufs4* KO mice until sacrifice at P51. NV354 delayed motor decline at P41 (Figures S7A and S7B), and counteracted weight loss (Figures S7D–S7F). However, there was no effect on clasping or body rotation (Figure S7C), distance or speed in an open field (Figures S7G–S7H), or breathing abnormalities (Figures S7I–S7J).

### Preventative effects of the succinate prodrug on metabolic decompensation and parkinsonian symptoms in rotenone-treated rats

**Figure 5:**
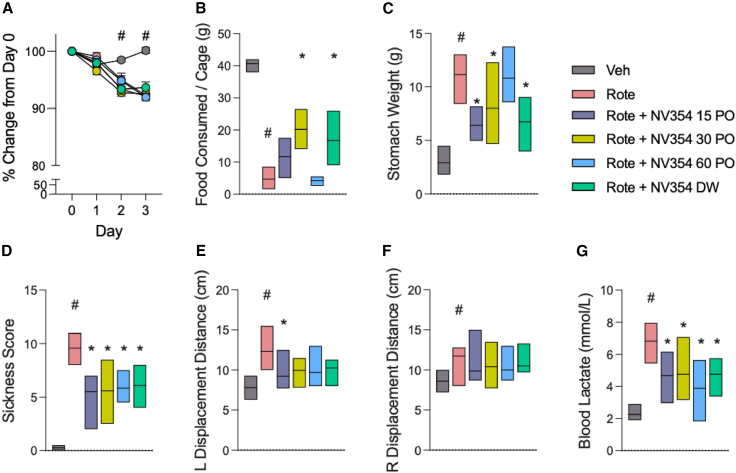
NV354 reduces Parkinson-like pathology and metabolic decompensation induced by complex I inhibition (A) Body weights relative to baseline (day 0) following daily treatment with vehicle (Veh) or rotenone (Rote; 2.75 mg/kg, IP), with or without oral NV354 administration (PO 7.5–30 mg/kg and DW 0.75 mg/mL), over a period of 4 days. (B) Average food consumption per cage at day 2–3 of rotenone treatment. (C) Impaired gastric emptying measured by increased average stomach weight at the end of rotenone treatment (day 3). (D) Decreased rotenone-induced toxicity in NV354-treated animals measured by the mean sickness scores on days 2 and 3. (E and F) Increased displacement of left limb (E) and right limb (F), indicating postural instability caused by rotenone treatment alone (*p* > 0.10 for Veh vs. Rote + NV354) on day 3. (G) Blood lactate levels as a measure of metabolic decompensation following rotenone treatment (day 3). #*p* < 0.05 for Rote vs. Veh, ∗*p* < 0.05 for Rote vs. Rote + NV354 groups by one-way ANOVA followed by Šídák’s posthoc test for multiple comparisons, *n* = 6. Bar graph values represent the mean ± SEM.

Next, the acute effects of NV354 on metabolic decompensation and motor dysfunction were investigated during the early stages of rotenone-induced PD in rats, before the occurrence of severe and irreversible neuronal damage. We evaluated the effect of orally administered NV354 on an established model of rotenone-induced CI-dysfunction model of PD, which recapitulates both non-motor and motor symptoms of the disease.^31^^,^^32^ To determine if NV354 could prevent metabolic impairment and PD-like symptoms, Lewis rats (Janvier, France) were treated with 2.75 mg/kg rotenone IP once daily for 4 days (days 0–3) and NV354 (7.5–30 mg/kg twice daily PO, or 0.75 mg/kg DW). Control rats received the same volume of vehicle. Rotenone treatment reduced body weight, food consumption, and gastric emptying, which became significant on days 2 and 3 (Figures 5A–5C). NV354 had no effect on body weight, but it improved food consumption and gastric emptying (*p* < 0.05 for Rote vs. Rote + NV354). Rotenone-treated animals also developed hallmark parkinsonian symptoms, such as bradykinesia, gait instability, and lethargy, which were quantified as part of a 14-point sickness scoring system (Figure 5D). NV354 prevented the onset of these symptoms on days 2–3 (*p* < 0.01 Rote vs. Rote + NV354). PD-related motor impairment was partially alleviated by NV354, with improved left limb displacement (Figure 5E*; ∗p* < 0.05 Rote vs. Rote + NV354; *p >* 0.10 for Veh vs. all doses Rote + NV354; Figure 5E) but no effect on right limb displacement (*p >* 0.80 for Rote vs. all doses Rote + NV354; Figures 5F and 5G) on day 3. To determine whether NV354 could attenuate metabolic decompensation induced by CI inhibition, blood lactate measures were quantified in rotenone-treated rats on day 3. NV354 reduced the elevation in blood lactate caused by rotenone toxicity (Figure 5G).

## Discussion

Succinate does not passively transport across cell membranes due to its charged nature. Therefore, to deliver succinate intracellularly, a carboxylic acid prodrug strategy is required. The initial attempt to generate cell-permeable succinate derivates resulted in a series of acyloxy alkyl esters of succinate that displayed high *in vitro* activity, resulting from high cell penetration, but poor *in vivo* stability.^20^ Thus, a series of other carboxylic acid prodrug approaches were assessed. One compelling approach was to assess succinyl thioesters. The concept was that not only would the compound be cell penetrant but could undergo one of two fates. First, it could be subject to standard carboxyl esterases to liberate the free acid (succinate), or it could undergo trans thio esterification with endogenous thiols to liberate metabolically active compounds (succinyl coenzyme A). *N*-acetylcysteamine thioesters, as mimics of the pantetheine chain on coenzyme A, have been used in biosynthetic studies to deliver free carboxylic acids into bacterial and fungal cells for the generation of the respective CoA thioester, and, so, this approach was adopted.^33^ NV354 was then selected from a series of compounds assessed for *in vitro* activity and favorable PK characteristics.

The results of this study demonstrated that the succinate prodrug NV354 prevents pathognomonic features of LS in *Ndufs4* KO mice and motor dysfunction caused by CI inhibition in a rat PD model. We have previously shown that respiratory dysfunction in patient-derived cells with CI deficiency can be corrected by providing succinate to cells via a prodrug strategy, feeding into the electron transport chain downstream of CI. Similar to PMD patient cells, mitochondria from *Ndufs4* KO and rotenone-treated animals are CI deficient, NADH metabolism is restricted, the function of CII is upregulated, and cellular succinate levels are decreased.^14^^,^^18^^,^^28^^,^^34^ Therefore, we hypothesized that facilitated intracellular delivery of succinate to animals with failing mitochondrial CI using a prodrug strategy may restore critical levels of succinate and its related intermediaries to cellular metabolism and regulation. Cell-permeable succinate prodrugs increase mitochondrial respiration linked to ATP production and tricarboxylic (TCA) cycle metabolite levels in cellular models of CI dysfunction.^20^^,^^35^ Further, succinate prodrugs may restore succinate supply for critical metabolic reactions via the TCA cycle, muscle remodeling and immune cell signaling via the succinate receptor 1 (SUCNR1),^36^^,^^37^ and stabilization of hypoxia-inducible factor-1 (HIF-1) via inhibition of prolyl hydroxylases.^38^^,^^39^ Thus, intracellular delivery of succinate may ameliorate the overall effects of CI deficiency and its secondary effects on ROS and succinate depletion, which may be responsible for the demonstrated positive effects of NV354 including body weight preservation, improved motor function, effects on whole-body respiration and thermal stress, reduced blood lactate levels, and neuroprotection.

Along with reduced ATP, CI impairment in LS and PD is associated with increased mitochondrial ROS production,^27^^,^^40^^,^^41^ which is pro-inflammatory and neurotoxic.^42^^,^^43^ Herein, we provide the first *in vivo* evidence of increased ROS production in the brain of an LS mouse model*.* NV354 prevented this increase, as well as downstream oxidative damages in the *Ndufs4* KO brain. In addition to generating oxygen radicals, brain mitochondria are powerful scavengers of ROS, depending on substrate supply and the intactness of the inner mitochondrial membrane.^44^^,^^45^ The limited substrate supply caused by CI deficiency may be partially restored by NV354 treatment, and this could impact the ability of mitochondria to scavenge ROS positively. The thiol group in NV354 may potentially also contribute to redox homeostasis. However, compounds with known antioxidant properties and positive effects on glutathione status have shown varied effects in LS models and mitochondrial disease patients.^30^^,^^46^^,^^47^^,^^48^ In support of this, we demonstrate that NV354 decreases DHE oxidation more effectively than SNAC alone in non-inhibited and CI-inhibited, but not in CIII-inhibited, human cells, which suggests that the reduction in ROS is specifically related to metabolism of the released succinate.

While the precise mechanisms responsible for the development of these brainstem lesions are unknown,^49^ collective evidence suggests they may be induced by CI impairment in VN glutamatergic neurons,^22^ which activate pro-inflammatory microglia in an ROS-dependent manner.^50^ In turn, the microglia promote neuronal death^25^ through a mechanism that may also involve ROS.^51^ This process may contribute to the loss of neurons and accumulation of hypertrophic microglia in the core of the lesion, with activated astrocytes surrounding the periphery, as we show in untreated *Ndufs4* KO mice. NV354 treatment increases neuronal survival and blocks gliosis, thereby precluding the emergence of hyperintense lesions on T2 weighted MRI. While MRI lesions may spontaneously reverse in the clinical LS population,^24^^,^^50^ we found no evidence of reversal regardless of treatment. None of the mice demonstrated lesions at ∼30 days, and those with MRI hyperintensity at follow-up also showed evidence of neuroinflammation and/or glial scarring in the lesion area at the end point by IHC. Interestingly, these lesions are thought to be directly causal of the early death from respiratory failure seen in the *Ndufs4 KO* mice, through faltering neurological control over the respiratory system.^15^^,^^16^ Despite the striking reduction of brainstem lesions in over 90% of NV354-treated animals analyzed through MRI, NV354 had no appreciable effect on *Ndufs4* KO breathing or mortality. Thus, our results suggest two major insights into LS-related pathology: (1) oxidative stress may be a key determinant of the pathognomonic MRI brainstem lesions, and 2) the VN lesions themselves are not solely responsible for the breathing abnormalities and mortality of *Ndufs4* KO mice. The latter is also supported by the partial effect on breathing and minimal extension of lifespan (20 days) under VN-specific gene therapy,^16^ compared with systemic therapies that have more than doubled the *Ndufs4* KO lifespan.^28^^,^^52^

NV354 also showed therapeutic potential in a rat model of rotenone-induced Parkinson’ disease, attenuating metabolic decompensation and partially alleviating the initial motor impairments. While we recognize that the 4-day rotenone treatment might not fully recapitulate the chronic neurodegenerative process observed in longer-term models, our experimental approach provides valuable insights into the early therapeutic potential of NV354 in mitigating rotenone-induced toxicity. Our choice of a shorter treatment duration aimed to minimize animal suffering and to focus on the acute effects of NV354 on metabolic decompensation and motor dysfunction before irreversible neuronal damage occurs. Further study of NV354 with a longer rotenone treatment period or transgenic PD model is required to fully reflect the chronic processes of PD. A second limitation in our rat study is that the drinking water-only (DW) group served as the primary control in these experiments. Specifically, the absence of a rotenone naive group receiving NV354 (Veh + NV354) makes it difficult to fully isolate the potential therapeutic effects of NV354 treatment, warranting further investigation. However, it is noteworthy that NV354 had a minimal effect on similar parameters (body weight and water consumption) in the WT mouse when provided orally (via gavage or drinking water).

In summary, despite the very short plasma half-life in rodents, the succinate prodrug NV354 protected against metabolic decompensation, oxidative stress, gastrointestinal impairment, motor dysfunction, neuroinflammation, and neuronal death caused by CI impairment in multiple animal models, however with non-uniform efficacy. While more research is required to determine the precise metabolic pathways involved, the protection conferred by NV354 suggests that succinate prodrugs hold therapeutic promise for diseases involving CI dysfunction. Lesion prevention without complete disease reversal highlights the complexity of LS pathology and strengthens the importance of further exploring the mechanism of action of NV354.

Although a strong correlation between NV354 treatment, ROS reduction, and lesion improvement was observed, further research is needed to definitively confirm the causative link. Future studies could explore this relationship using a variety of approaches, including genetic manipulation of ROS-related pathways in VN neurons or targeted antioxidant delivery, with longitudinal imaging of ROS and lesion development. Other mechanisms such as the restoration of mitochondrial function and energy production may also contribute to the observed effects.

### Limitations of the study

Our findings demonstrate a strong correlation between NV354 treatment, reduction of ROS levels, and amelioration of CNS lesions. However, further experiments, such as genetic manipulation of ROS-related pathways, are required to establish causality. Our study’s 4-day rotenone treatment offers early insights into NV354’s therapeutic potential against rotenone-induced toxicity, but may not fully replicate chronic neurodegeneration. This shorter duration was chosen to minimize animal suffering and focus on acute effects, necessitating further research with longer treatments to fully understand chronic disease processes. Additionally, using the DW group as the primary control limits our ability to isolate NV354’s specific therapeutic effects in rats, highlighting the need for more comprehensive investigations.

## Resource availability

### Lead contact

Requests for further information and resources should be directed to and will be fulfilled by the lead contact, Dr. Meagan J. McManus (mcmanusmj@arizona.edu).

### Materials availability

This study did not generate new unique reagents.

### Data and code availability

• All data reported in this article are available within the article and its supplemental files. Further data inquiries can be directed to the lead contact.
• No original code was generated in this study.
• Any further information can be obtained from the lead contact upon request.

## Acknowledgments

This work was supported by the U.S. Department of Defense grant PR171698 awarded to T.K., 10.13039/100000065National Institute of Neurological Disorders and Stroke grant R01NS114656 awarded to M.J.M., AEI Proyectos I+D+i PID2020-114977RB-I00 awarded to A.Q., BES-2015-073041 awarded to P.P.D., PRE2018-083179 awarded to L.S.B., and RYC2019-028501-I awarded to E.S.

## Author contributions

Funding, M.J.M., P.P.D., L.S.B., E.S., A.Q., and T.J.K.; conceptualization, methodology, resources, validation, and writing – review & editing, M.J.M., D.C.W., S.P., C.A., M.K., J.K.E., M.J.H., E.E., A.Q., and T.J.K.; data curation, investigation, and formal analysis, M.J.M., Y.Z., C.A., N.K., P.P.D., L.S.B., E.S., I.Y., L.R., M.S., W.J.M., A.R., J.M., N.D., A.G., S.P., S.J.M., and L.W.; project administration and resources, M.J.M., A.Q., and T.J.K.; supervision and validation, M.J.M., M.J.H., E.E., A.Q., and T.J.K.; visualization, M.J.M., E.E., A.Q., and T.J.K.; writing – original draft, M.J.M.

## Declaration of interests

A.G., M.K., M.J.H., E.E., and J.K.E. receive or have received salary support and/or travel reimbursements and/or grants from Abliva AB, a Swedish public company developing pharmaceuticals in the field of mitochondrial medicine. M.J.H. and E.E. are currently Abliva employees and members of its management team. S.J.M. acts and L.W. has acted as paid consultant to Abliva AB. Abliva AB has filed patents related to succinate prodrugs, some of which have A.G., M.J.H., E.E., and J.K.E. as named inventors (relevant US patents are listed below):

Succinate prodrug, compositions containing the succinate prodrug and uses thereof (US20230033294-A1, 11565998-B2, and 20220162162-A1); novel cell-permeable succinate compounds (US20210401792-A1 and 20170105961-A1); cell-permeable succinate compounds (US11147789-B2); succinate prodrugs for use in the treatment of lactic acidosis or drug-induced side effects due to complex I-related impairment of mitochondrial oxidative phosphorylation (US10307389-B2 and 20170100359-A1); protected succinates for enhancing mitochondrial ATP production (US9670175-B2 and 20150259317-A1); prodrugs of succinic acid for increasing ATP production (US20170105960-A1).

## STAR★Methods

### Key resources table

**Table:** 

REAGENT or RESOURCE	SOURCE	IDENTIFIER
**Antibodies**		

Mouse monoclonal anti-NeuN	Abcam	Cat# ab104224; RRID:AB_10711040
Rabbit polyclonal anti-NeuN	Thermo Fisher	Cat# PA5-78499; RRID:AB_2736206
Mouse monoclonal anti-GFAP	Abcam	Cat# ab5804; RRID:AB_2109645
Goat anti-mouse Alexa Fluor 488	Thermo Fisher	Cat# A11008; RRID:AB_143165
Goat anti-rabbit Alexa Fluor 488	Thermo Fisher	Cat# A11001; RRID:AB_2534069
Goat anti-mouse Alexa Fluor 568	Thermo Fisher	Cat# A11004; RRID:AB_2534072
Goat anti-rabbit Alexa Fluor 568	Thermo Fisher	Cat# A11011; RRID:AB_143157

**Chemicals, peptides, and recombinant proteins**		

NV354	Isomerase Therapeutics	N/A
[13C4]NV354	Isomerase Therapeutics	N/A
Dihydroethidium (DHE)	Thermo Fisher	D11347
MitoSOX Deep Red	Thermo Fisher	M36008
Rotenone	Sigma-Aldrich	R8875
Antimycin A	Sigma-Aldrich	A8674
Hoechst 33342	Thermo Fisher	H3570
OxiSelect Nitrotyrosine ELISA Kit	Cell Biolabs	STA-305
MDA-Protein Adduct ELISA Kit	Cell Biolabs	STA-332

**Experimental models: Organisms/strains**		

Ndufs4 knockout mice	Palmiter Lab	N/A
CD1 mice	Charles River	N/A
Sprague Dawley rats	Charles River	N/A

**Software and algorithms**		

Fiji (ImageJ)	NIH	https://imagej.net/software/fiji/downloads
Ethovision XT	Noldus	N/A
FlowJo v10	BD Biosciences	N/A
GraphPad Prism v6.0–9.0	GraphPad Software	N/A
MATLAB	MathWorks	N/A
Living Image Software	Perkin Elmer	N/A
eDacq v1.9.0	EMMS	N/A

**Other**		

Alzet osmotic mini-pump (Model 1004)	Durect Corporation	N/A
IVIS Spectrum	Perkin Elmer	N/A
7T MRI scanner with Bruker gradient	Bruker	N/A
CLAMS metabolic cages	Columbus Instruments	N/A

### Experimental model and study participant details

#### Animal models

The aim of this study was to examine the therapeutic potential of the succinate prodrug NV354 for neurodegenerative diseases involving mitochondrial complex I dysfunction, using several animal models. Ndufs4 (+/-) mice from the Jackson Laboratory were bred to produce WT and KO mice, which were investigated from 0-6 mo. of age. Both sexes were utilized in the study, but sex was not incorporated as a co-variable due to limited sample size. Male CD1 mice (Charles River), aged 8-10 weeks, were utilized for pharmacokinetic (PK) profiling due to their suitability for serial sampling. Male Sprague Dawley rats (7-9 weeks old, weighing 269-300 g) purchased from Janvier Labs were employed in a rotenone-induced Parkinson’s disease (PD) model.

All animals were housed in a controlled environment (12 h light/12 h dark condition) with ad libitum access to food and water. Each experimental protocol was approved by the institutional animal care and use committee (IACUC) at the Children’s Hospital of Philadelphia Research Institute, the regional animal experimental ethics committee in Stockholm, or the Ethics Committee of the Universitat Autònoma de Barcelona. All efforts were made to minimize both suffering and the number of animals used in the experiments. The potential impacts of sex on the findings in animal models were not explored, warranting caution in generalizing results across sexes.

#### Human samples

The study was approved by the Swedish Ethical Review Authority (2019-04947). Anonymized leukocyte concentrates (n=7) were obtained under permit number 2022:23 from whole blood processed using the Reveos 3C System® (Terumo Blood and Cell Technology, USA) at the Clinical Immunology and Transfusion Medicine Department, Skåne University Hospital, Lund, Sweden. Due to the anonymization of samples, information regarding age, sex, gender, ancestry, race, or ethnicity was not available. However, it is estimated that more than 50% of blood donors in Sweden are female, and with certain age restrictions in place, the analyzed samples are likely from healthy individuals aged 18-65 years, including both females and males. Samples were randomly allocated to experimental groups. As the magnitude of change in the evaluated parameter (mean fluorescence intensity, MFI) was not pre-defined, power calculations for sample size were not applied.

### Method details

#### Compound synthesis and characterization

NV354 (methyl 3-[(2-acetylaminoethylthio)carbonyl]propionate) was synthesized by coupling *N*-acetylcysteamine with monomethyl succinate in the presence of 1,1′-carbonyldiimidazole. For the synthesis of [^13^C_4_]-labeled NV354, [^13^C_4_]3,4-dihydro-2,5-furandione was first prepared by dehydration of [^13^C_4_]succinic acid using trifluoroacetic anhydride (TFAA) under reflux. The resulting intermediate was then subjected to solvolysis in refluxing methanol to generate [^13^C_4_]monomethyl succinate. This compound was subsequently coupled with *N*-acetylcysteamine (SNAC) in the presence of 1,1′-carbonyldiimidazole to yield [^13^C_4_]NV354 ([^13^C_4_]methyl 3-[(2-acetylaminoethylthio)carbonyl]propionate).

The aqueous solubility and stability of NV354 were evaluated across different media. Samples were centrifuged at 13,000 rpm for 15 minutes, and the resulting supernatant was analyzed by high-performance liquid chromatography (HPLC). NV354 concentration was determined by measuring absorbance at 230 nm and comparing it to a standard curve generated using NV354 dissolved in DMSO.

#### Pharmacokinetics and bioavailability

NV354 is a prodrug of the tricarboxylic acid (TCA) cycle intermediate succinate, conjugated to an *N*-acetylcysteamine (SNAC) carrier to facilitate intracellular delivery upon metabolism. Due to its rapid degradation in rodent plasma, conventional pharmacokinetic (PK) profiling based on parent compound blood levels is not informative. Instead, two complementary in vivo PK strategies were employed: (1) quantification of the SNAC moiety as a proxy for NV354 metabolism, and (2) detection of [^13^C]-labeled succinate and its downstream metabolites following administration of [^13^C_4_]NV354.

All NV354 formulations were prepared in phosphate-buffered saline (PBS, pH 7.4), unless otherwise specified. For mouse PK studies, male CD1 mice received either intravenous (IV) injection (50 mg/kg) or oral gavage (PO, 200 mg/kg) of [^13^C_4_]NV354 (n = 3 per group). Serial venous blood samples were collected at 0.25, 1, 2, and 4 hours post-administration, and whole blood was analyzed for [^13^C_4_]succinate levels. In a dose-response study, male CD1 mice (23–25 g) were administered increasing oral doses of [^13^C_4_]NV354 (0–90 mg/kg), and sacrificed at 15 minutes post-dose for analysis of [^13^C_4_]succinate and SNAC in both plasma and brain tissue (n = 3 per dose group). To evaluate tissue distribution, additional CD1 mice were given 200 mg/kg PO of [^13^C_4_]NV354, while control mice received an equimolar dose of [^13^C_4_]succinate (100 mg/kg PO). Metabolite concentrations were measured in major organs 15 minutes post-dosing.

Although C57BL/6 mice were used in behavioral studies, CD1 mice were selected for PK profiling due to their suitability for serial sampling. Notably, Barr et al.^53^ demonstrated that while some inter-strain differences exist, pharmacokinetic parameters show strong concordance across mouse strains—including C57BL/6 and CD1—supporting their interchangeability in early PK screening when interpreted appropriately.

For rat studies, male Sprague Dawley rats (7–9 weeks old, 269–300 g) received an IV dose of [^13^C_4_]NV354 (20 mg/kg) and were sacrificed at multiple time points (5 min to 2 h) for analysis of [^13^C_4_]succinate in plasma and tissues. To determine NV354’s plasma half-life (t_1_/_2_), the compound was incubated in mouse, rat, and human plasma, and quantified at time points 0, 5, 15, 30, 45, and 60 minutes using mass spectrometry. The analyte was detected via tandem MS (MS-MS), using imipramine as an internal standard and quantifying a selected daughter ion.

#### Drug administration protocols

In mice, NV354 treatment commenced at the time of weaning (postnatal day 19). The compound was administered either via oral gavage (PO; 0, 50, 250, 500, or 750 mg/kg once daily) or in the drinking water (DW; 250 mg/kg/day) to both wild-type (WT) and Ndufs4 knockout (KO) animals. Due to impaired motor function, Ndufs4 KO mice were cohoused with WT or heterozygous littermates and provided with moist chow placed on the cage floor, along with hydrogel supplemented with or without NV354. Upon progression of motor symptoms (∼postnatal day 45), NV354 was also delivered via daily subcutaneous injection to compensate for reduced water intake. Control animals received subcutaneous saline.

In a separate experimental cohort, Ndufs4 KO mice were treated with either vehicle (1:1 DMSO:PEG 400, *n* = 11) or NV354 (70 mg/kg/day, *n* = 10) delivered continuously for 4 weeks via subcutaneously implanted osmotic mini-pumps (Alzet model 1004, Durect Corporation, Cupertino, CA; 0.11 μL/h infusion rate), beginning on postnatal day 23 and continuing until sacrifice on day 51. Blinded clinical assessments were performed every 3–5 days, with motor and respiratory function monitored at 10-day intervals.

In rats, NV354 was administered twice daily via oral gavage at doses of 7.5, 15, or 30 mg/kg, or vehicle (water; 10 mL/kg), for four consecutive days at 6 ± 1 hour intervals. The first NV354 dose was co-administered with rotenone or vehicle via intraperitoneal injection. In an additional group, NV354 was provided in drinking water (0.75 mg/mL), which was refreshed daily.

#### Health and behavioral monitoring

The impact of NV354 on lifespan and healthspan in Ndufs4 KO mice was assessed through daily monitoring of phenotypic progression, including evaluations of ataxia, body weight, and surface temperature. A composite ataxia sickness scoring system was adapted from Quintana et al. ^(^^15^^)^, capturing key features of Ndufs4 KO motor impairment. Scoring was conducted on a 0–1 scale for each of the following categories:

• Trunk curl/hind-limb clasping: Scored as 1 for hind limb clasping or “helicoptering” behavior (rotational movement due to weakness), observed during a 1-minute tail suspension test.
• Limb tone: Scored as 0.5 per hind limb displaying marked resistance or freezing.
• Tremor: Scored as 1 for any tremor observed during the 1-minute observation period.

All behavioral assessments were performed by investigators blinded to treatment groups.

The Open Field Test was used to evaluate anxiety-related behavior and motor function in a novel environment. Experimenters were blinded to the treatment status of the animals. Animals were placed in an empty arena (43.2 x 43.2 cm transparent acrylic walls and white floor) and their position monitored and recorded by Ethovision tracking software (Ethovision XT, Noldus; Wageningen, The Netherlands) over a period of 15 min. Total distance traveled, velocity, bouts of immobility, and vertical rears provide the assessment of motor function. Body contour and the amount of time spent close to the wall (thigmotaxis) versus the amount of time spent in, and latency to, the center zone indicates the relative anxiety level of the mouse.^54^

Motor coordination was assessed using the Rotarod test (LE8205, PanLab). Mice underwent a 30-minute habituation session prior to testing. Each testing session consisted of five trials per animal, with a 6-minute inter-trial interval. During each trial, mice were placed on an accelerating rod (4 to 40 rpm over 3 minutes), and the latency to fall was recorded.

#### Metabolic studies

The Comprehensive Lab Animal Monitoring System (CLAMS; Columbus Instruments, Columbus, OH) was utilized to track the effect of NV354 treatment on metabolism through the progression of mitochondrial disease in *Ndufs4* KO mice. Mice were singly housed in the metabolic cages, which were fitted with running wheels, and water and food ad libitum. During testing, the room was maintained on a 12:12 light: dark cycle. The CLAMS metabolic chambers were equipped with sensors to measure respiration frequency, the volume of oxygen consumption (VO_2_) and carbon dioxide production (VCO_2_), respiratory exchange ratio (RER), and heat production using an airflow of 600 mL/min at 22°C. Mice were thermally challenged in 30°C for 6 h during the dark cycle.^55^

#### Plethysmography

Unrestrained whole-body plethysmography (EMMS) was performed by placing a mouse in a 90 mm pre-calibrated chamber for 45 min to allow acclimation, followed by 15 min of experimental period. Respiratory frequency and tidal volume per body weight (μL/g) were measured by v1.9.0 eDacq software (ESS101A, EMMS).

#### In vivo neuroimaging

To prepare for fluorescence imaging, hair was removed from the scalp region of mice using Nair hair removal lotion at least 48 hours prior to imaging. Mice were then injected intraperitoneally (IP) with dihydroethidium (DHE; 20 mg/kg) and imaged 24 hours later using the In Vivo Imaging System (IVIS) Spectrum (Perkin Elmer, Waltham, MA, USA). A spectral unmixing (SPUM) protocol was applied to distinguish the fluorescent signal from oxidized DHE (2-OH-E^+^ and E^+^)^56^^,^^57^ from background tissue autofluorescence (TAF). This approach compensates for spectral overlap between oxidized DHE (excitation/emission: 500/580 nm) and the broad-spectrum autofluorescence inherent to mouse tissue. Using multiple excitation/emission filter sets, the SPUM algorithm deconvoluted signals based on reference spectra from untreated (TAF-only) and DHE-treated control animals. These spectra were stored in a spectral library and applied consistently across all imaging sessions using identical acquisition parameters. Identical regions of interest (ROIs) were defined over the brain, and total radiant efficiency was quantified using Living Image software (Perkin Elmer).

For magnetic resonance imaging (MRI), mice were scanned at approximately postnatal days 30 and 55 using a 7-Tesla horizontal bore magnet equipped with a 12 cm, 65 G/cm gradient insert (Bruker, Ettlingen, Germany). Anesthesia was maintained with 0.1–2% isoflurane in pure oxygen. A four-element (∼3 cm) surface coil was used for signal reception, and an 86 mm volume coil for excitation. Breathing rate was monitored using an MRI-compatible physiological monitoring system (SA Instruments, Stony Brook, NY, USA), and body temperature was maintained at 37°C using a circulating water bath. Three-dimensional fast spin-echo images were acquired with the following parameters: repetition time (TR) = 7 ms, echo time (TE) = 90 ms, RARE factor = 8, in-plane resolution = 125 μm, and slice thickness = 500 μm.

MRI images were independently reviewed by a pediatric neuroradiologist blinded to treatment group, with expertise in mitochondrial disorders. Lesions were characterized by their anatomic location (olfactory bulb, cortex, white matter, deep gray nuclei, and cerebellum) and T2-weighted signal intensity. Lesions were further classified as mild or severe based on two criteria: (1) signal homogeneity, homogeneous lesions indicating edema vs. heterogeneous lesions suggesting necrosis, liquefaction, or hemorrhage; and (2) presence of tumefactive features—defined as lesion expansion beyond the normal anatomical boundaries and distortion of surrounding structures.

#### Immunohistochemistry

All mouse brains were fixed in 4% paraformaldehyde (PFA) immediately after extraction, post-fixed for at least 24h at 4°C and embedded. Paraffin-embedded brainsections (10 μm) were deparaffinized and incubated with antigen retrieval buffer (citrate buffer) at 98∼100°C for 30 min. Fifteen min of 0.5% Triton X-100 incubation were applied to permeabilize tissues. Subsequently, all slides were put into Sequenza Immunostaining Center (Fisher Scientific, 73-310-017) and incubated with 100 mM Glycine for 10 min. After rinse with PBS, the slides were blocked with either 10% bovine serum albumin (BSA) or Mouse on Mouse (M.O.M) IgG Blocking reagent (1:25, PBS, Vector Laboratories,101098-256) for 1 h at RT, followed by a wash of 1% BSA in PBS for 5 min, and incubated with primary antibodies overnight at 4˚C (key resources table). The following day, selected slides were incubated with first secondary antibodies (key resources table) for 1 h at room temperature and rinsed with 1% BSA in PBS three times. Then, selected second primary antibodies were incubated for 1 h at room temperature, followed by another wash of 1% BSA in PBS. The corresponding second secondary antibodies (key resources table) were then applied to selected slides and incubated for 1 h at room temperature, counterstained with Hoechst (1:2000, PBS, Thermo Fisher Scientific, H3570), and mounted with VectaShield Antifade Mounting Media (Vector Laboratories, 101098-042). Images were acquired by using Zeiss Axio Observer Microscope (Jena, Germany). Images were uploaded onto Fiji (ImageJ) where an ROI was drawn around the vestibular nuclei (VN) and applied to all brains. Fluorescence intensity (arbitrary units, AU) for GFAP (brainstem) and Iba-1 (lesion area) was quantified using Fiji, following methods described in Gallagher et al.^58^ As a measure of microglial activation, the soma size of microglia within the lesion, or in the anatomically corresponding region in control mice, was manually traced and quantified using Fiji as described in Zhu et al.^59^ For neuronal counts, NeuN staining was changed to white using Zen Software (Jena, Germany) to maintain consistency. Neurons within the VN ROI were counted in Fiji using the Cell Counter plugin (Plugins à Analyze à Cell Counter). Images were 2.02 mm x 1.52 mm, and neuron counts were normalized by area (neurons/mm^2^).

#### ELISA assays

Nitrotyrosine adducts were quantified by enzyme-linked immunosorbent assay (ELISA) using brain tissue homogenate according to the OxiSelect Nitrotyrosine ELISA Kit protocol (STA305, Cell Biolabs, San Diego, CA, USA). Lipid peroxidation was assessed by quantification of malondialdehyde (MDA)-protein adducts (MDA-protein-adduct ELISA Kit, Cell Biolabs Inc., San Diego, USA) according to the manufacturer’s protocol.

#### Flow cytometry

MitoSOX Deep Red and dihydroethidium (DHE)-based flow cytometry were used to detect mitochondrial reactive oxygen species (ROS) and cytoplasmic ROS, respectively. Human peripheral blood mononuclear cells (PBMCs) were isolated from whole blood as previously described^60^ and stained with the intracellular fluorochromes MitoSOX Deep Red (20 μM) or DHE (50 μM) for 20 min at 37°C. PBMCs were then treated with either rotenone (2 μM), antimycin A (AMA;1 mg/mL), or DMSO for 15 min before addition of the succinate prodrug NV354 (100 μM), its cleaved by-product N-acetylcysteamine (SNAC) (100 μM), or DMSO. The BD LSRII flow cytometer allows for the measurement of MitoSOX Deep Red and DHE fluorescence upon excitation with the Blue laser (488 nm) and detection with the 665/30 and 610/20 bandpass filters. Lymphocytes were gated based on forward and side scattering and mean fluorescence intensity (MFI) values were compared using FlowJo software v. 10 (BD Biosciences).

#### Rotenone rat model and neurobehavioral assessment

Rats were administered rotenone or vehicle once daily for four consecutive days (Day 0 to Day 3) via intraperitoneal (IP) injection. Rotenone was delivered at a dose of 2.75 mg/kg in a vehicle solution composed of 98% Miglyol and 2% DMSO (1 mL/kg). To assess the therapeutic potential of NV354, rats were concurrently treated with increasing doses of NV354—administered either via oral gavage twice daily (7.5, 15, or 30 mg/kg) or in drinking water (0.75 mg/mL, refreshed daily).

Upon arrival, all animals were weighed and allowed to acclimate for one week prior to the start of treatment. During the study, body weight was recorded daily, and general health was assessed twice per day. Food and water intake were measured at the cage level at the same time each day. Rotenone-induced sickness was evaluated twice daily by a blinded observer using a 0–2 point scale, assessing the presence or severity of the following phenotypic features: piloerection, lethargy, bradykinesia, gait instability, porphyria, hypothermia, and kyphosis.

Venous blood samples were collected following the final behavioral test (Day 3). Blood was immediately analyzed for lactate, pH, and blood gas parameters using the VetScan iSTAT-1 Analyzer (Abaxis, USA). Plasma samples were stored at −20°C and later analyzed within 5 weeks for standard clinical chemistry markers using the VetScan VS2® system (Abaxis, USA). After blood collection, rats were euthanized with an overdose of pentobarbital. Stomachs were excised and weighed to assess gastric emptying. The gastrointestinal tract was examined macroscopically for abnormalities, and brains were harvested for post-mortem histological and molecular analyses.

On Day 3, postural instability was assessed in a blinded manner using a previously described “forelimb displacement” method.^56^^,^^57^ Briefly, animals were positioned vertically in a wheelbarrow-like posture on a tabletop covered with P-120 sandpaper, marked at 1cm intervals. While one forelimb remained planted on the surface, the animal’s center of mass was slowly shifted forward until the unrestrained forelimb initiated two “catch-up” steps to maintain balance. The distance from the original nose position to the point of the second step was recorded. Each forelimb was tested in three independent trials. All behavioral assessments were performed by investigators blinded to treatment groups.

### Quantification and statistical analysis

All statistical analyses were performed using MATLAB or GraphPad Prism (v6.0–9.0). Survival curves were compared using Mantel-Cox logrank test. Longitudinal data were analyzed by two-way ANOVA or mixed-effects models with Holm-Šídák correction. One-way ANOVA followed by Šídák's post-hoc test was applied to single time-point data for comparisons among three or more groups. Unpaired t-tests were used to compare two unpaired groups with normally distributed data, and Wilcoxon matched pairs signed rank test was used for paired, nonparametric data. Statistical significance was set at p < 0.05. Detailed statistical methods are described in Figure Legends.
