# Supplementary material for: The succinate prodrug NV354 prevents brain lesions and late-stage motor dysfunction in mitochondrial complex I deficiency
Source: iScience. 2026 Jan 16;29(2):114717. doi: 10.1016/j.isci.2026.114717 (PMC12907114; doi:10.1016/j.isci.2026.114717)
Supplement: Document S1. Figures S1–S7 and Tables S1 and S2 [file mmc1.pdf]

iScience, Volume *29*

Supplemental information

The succinate prodrug NV354 prevents brain lesions and late-stage motor dysfunction in mitochondrial complex I deficiency

Meagan J. McManus, Yi Zhu, Cesar Alves, Neha Kohli, Patricia Prada-Dacasa, Laura Sanchez-Benito, Elisenda Sanz, Irene Yee, Lozen Robinson, Malkah Sheldon, Walter J. McHugh, Abhay Ranganathan, Jennie Meng, Nina Duncan, Alvar Grönberg, Douglas C. Wallace, Sarah Piel, Michael Karlsson, Steven J. Moss, Lee Webster, Magnus J. Hansson, Eskil Elmér, Johannes K. Ehinger, Albert Quintana, and Todd J. Kilbaugh

Compound	$[C_4]$succinate Concentration (ng/mL, ng/g)						
	Plasma		Brain	Eyes	Liver	Heart	Muscle
	0.25 hr	0.5 hr	0.5 hr	0.5 hr	0.5 hr	0.5 hr	0.5 hr
**$[^{13}C4]$NV354**	954	1523	359	208	1957	3097	156
**$[^{13}C4]$Succinate**	3640	3253	18.1	30.3	996	1342	76.8

Compound	$[C_4]$succinate Concentration (μM)						
	Plasma		Brain	Eyes	Liver	Heart	Muscle
	0.25 hr	0.5 hr	0.5 hr	0.5 hr	0.5 hr	0.5 hr	0.5 hr
**$[^{13}C4]$NV354**	8	13	3	2	17	26	1
**$[^{13}C4]$Succinate**	31	28	0.2	0.3	8	11	1

Table S1. Comparison of succinate delivery to target organs in mice following continuous IV infusion of $^{13}C[C_4]$ NV354 *vs.* $^{13}C[C_4]$succinate.

Dosing		Blood	Plasma	Brain	Heart	Liver	Muscle
		[13C]succinate (ng/mL, ng/g)					
**$^{13}C[C_4]$NV354 PO 200 mg/kg (860 µmol/kg)**	Mean	12080	3383	1825	3263	16167	2650
	SD	4023	1839	1215	2479	6726	987
**$^{13}C[C_4]$NV354 IP 200 mg/kg (860 µmol/kg)**	Mean	14267	4880	1467	2020	12970	1903
	SD	1443	1025	264	318	4220	510
**$^{13}C[C_4]$succinate PO 100 mg/kg (850 µmol/kg)**	Mean	323	706	6.05	34.5	848	59.9
	SD	120	302	2.71	10.5	225	12.8
		SNAC (ng/mL)					
**$^{13}C[C_4]$NV354 PO 200 mg/kg (860 µmol/kg)**	Mean	33350	85930	14473	5027	9672	12177
	SEM	10669	28645	6787	3632	3622	6845
**$^{13}C[C_4]$NV354 IP 200 mg/kg (860 µmol/kg)**	Mean	44637	108733	19103	5895	15277	14193
	SEM	7954	6697	1724	2668	5425	1017
		[13C]succinate (µM)					
**$^{13}C[C_4]$NV354 PO 200 mg/kg (860 µmol/kg)**	Mean	101.5	28.4	15.3	27.4	135.9	22.3
	SEM	19.7	9.0	5.9	12.1	32.9	4.8
**$^{13}C[C_4]$NV354 IP 200 mg/kg (860 µmol/kg)**	Mean	120.9	41.4	12.4	17.1	109.9	16.1
	SEM	7.1	5.0	1.3	1.6	20.6	2.5
**$^{13}C[C_4]$succinate PO 100 mg/kg (850 µmol/kg)**	Mean	2.7	6.0	0.1	0.3	7.2	0.5
	SEM	0.6	1.5	0.0	0.1	1.1	0.1
		SNAC (µM)					
**$^{13}C[C_4]$NV354 PO 200 mg/kg (860 µmol/kg)**	Mean	279.8	720.9	121.4	42.2	81.1	102.2
	SEM	51.7	138.7	32.9	17.6	17.5	33.2
**$^{13}C[C_4]$NV354 IP 200 mg/kg (860 µmol/kg)**	Mean	374.5	912.2	160.3	49.5	128.2	119.1
	SEM	38.5	32.4	8.4	12.9	26.3	4.9

Table S2. Blood and organ concentrations of [13C]-labeled NV354, or equimolar [13C]-labeled succinate, and SNAC after 0.25 h of a single dose via IP or PO administration.

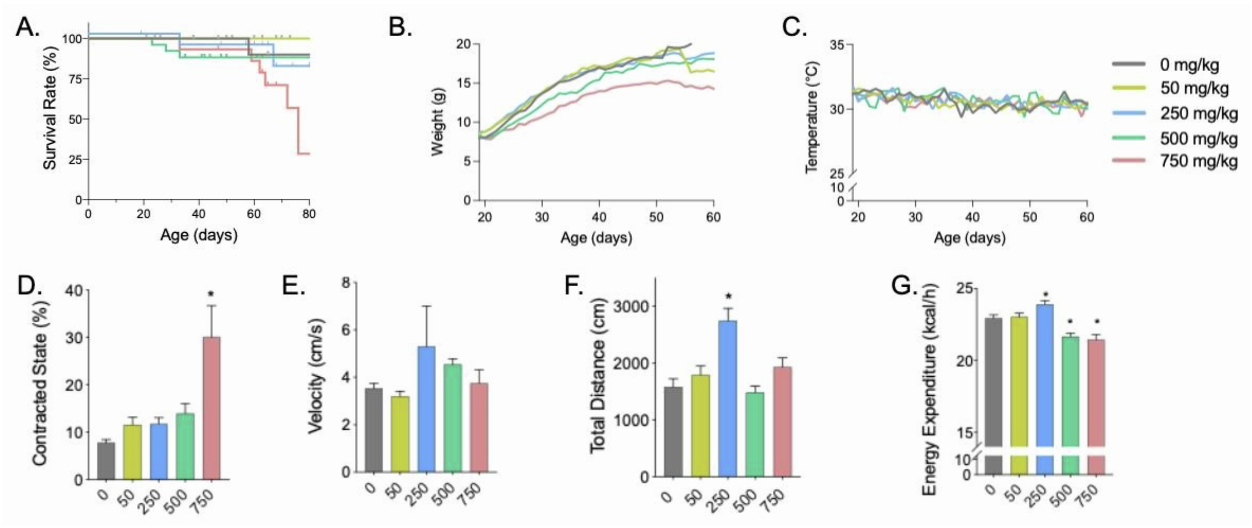

Figure S1. Dose optimization in C57B6/J mice administered NV354 via daily per oral (PO) for approximately 60d. A) Decreased survival and weight (**B**) at only the highest dose (750 mg/kg; P < 0.01 vs. baseline) by Mantel-Cox log-rank test, and no effect on surface body temperature (**C;** P > 0.10 for all doses vs. baseline) n=15-19. **D**) Contracted body contour (percent time) was increased at the highest dose and there was no change in velocity (**E)** in the open field test. **F**) Total distance traveled in running wheels and energy expenditure (**G**) was measured in metabolic cages during the dark cycle (n=3-10). *P < 0.05 vs. baseline by one-way ANOVA followed by Šídák's posthoc test for multiple comparisons. Values represent mean ± SEM.

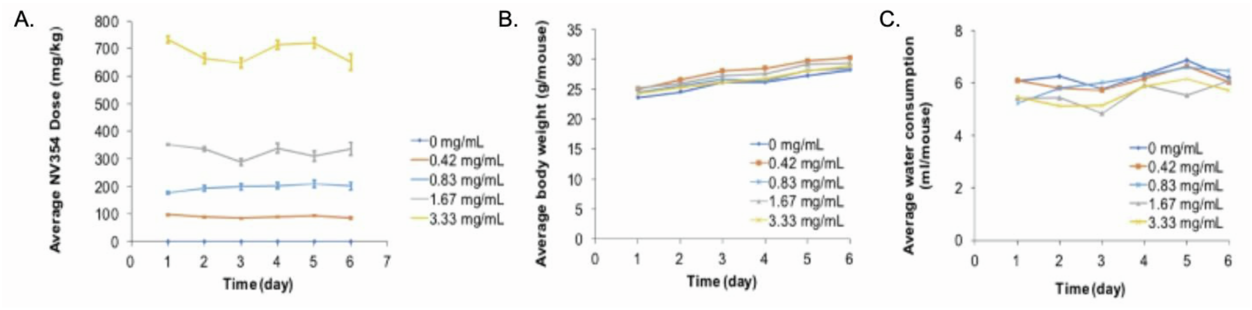

Figure S2. **Tolerability of increasing concentrations of NV354 administered in mice via the drinking water.** (**A**) Calculated daily doses ranging from 85 mg/kg to 746 mg/kg. (**B**) Average body weight per day over the 7-day dosing regimen. (**C**) Average daily water consumption. Analyzed by two-way ANOVA. Values represent mean ± SEM.

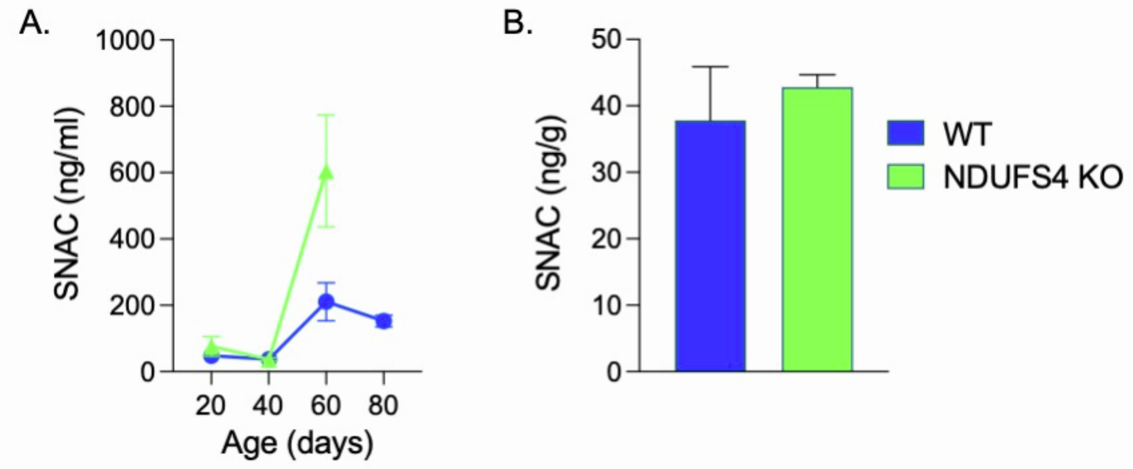

Figure S3. **Concentration of the NV354 metabolite SNAC in WT and *Ndufs4* KO mice treated with NV354 in the drinking water. A)** Plasma SNAC levels over 20-60d of treatment (P = 0.38). **B)** Brain SNAC levels at approximately 60d (P = 0.47) by one-way ANOVA followed by Šídák's posthoc test for multiple comparisons. Values represent mean ± SEM.

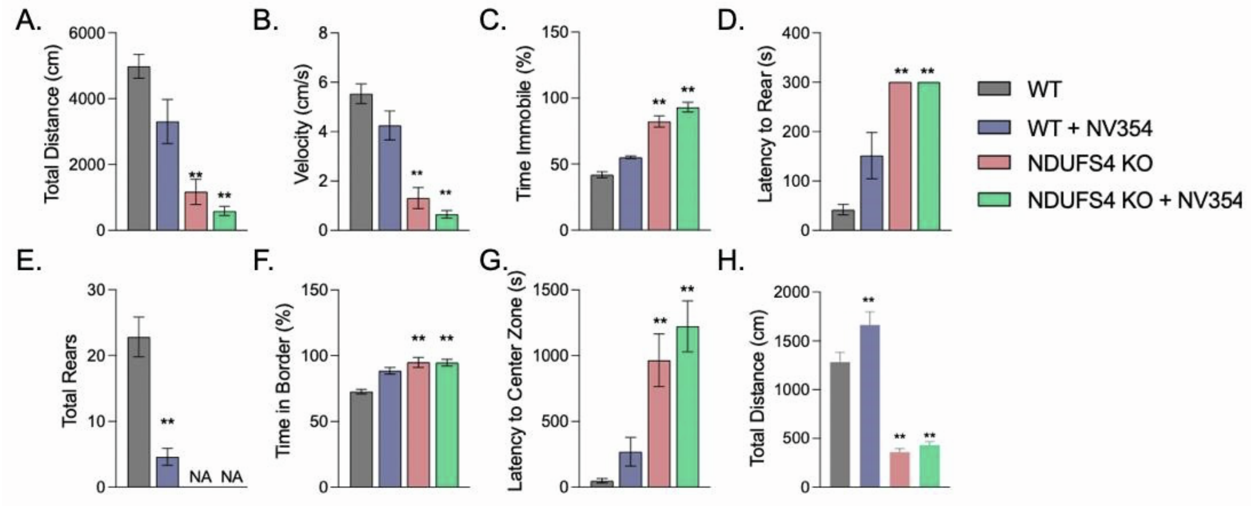

Figure S4. **NV354 did not alter behavior of *Ndufs4* KO mice in the open field test or home cage at 30d of age. A-E**) Leigh-like motor impairment in *Ndufs4* KO mice is shown by decreased total distance traveled (**A**) and velocity (**B**), increased time spent immobile (**C**), and the absence of rearing (**D-E**). **F-G**) *Ndufs4* KO mice appeared more anxious with increased thigmotaxis (**F**; time in the border) and increased latency to enter the center zone (**G**). **H)** NV354-treatment (DW, 250 mg/kg/day) increased activity of WT mice in the home cage environment, but had no effect in

Ndufs4 KO mice. $P > 0.05$ for *Ndufs4* KO + NV354 *vs. Ndufs4* KO, for all measures. $^{**}P < 0.001$ relative to WT by one-way ANOVA followed by Šídák's posthoc test for multiple comparisons. Values represent mean ± SEM, n = 6-16.

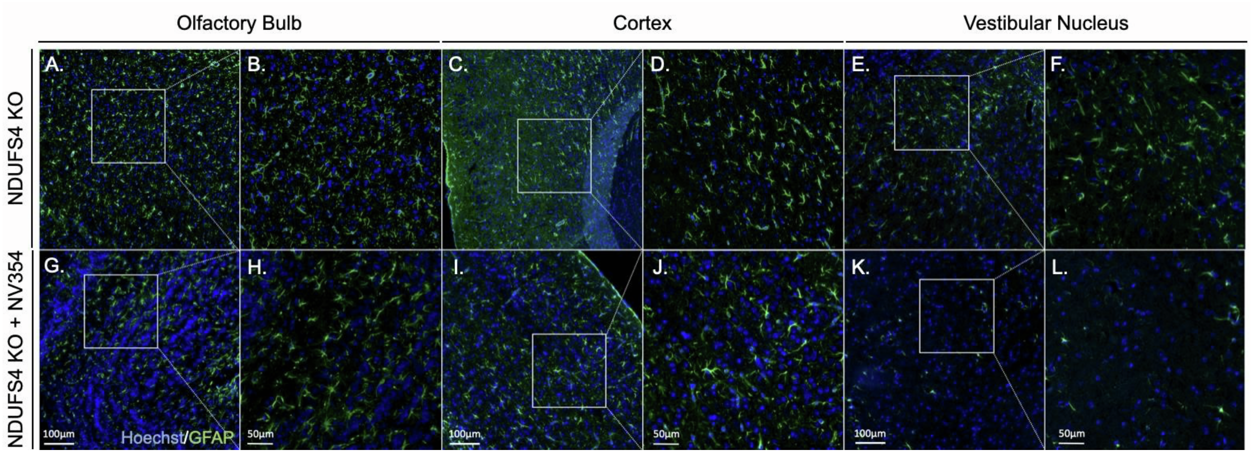

Figure S5. **NV354 prevents astrocyte proliferation in key regions of the *Ndufs4* KO brain. A-F** Activation of astrocytes (GFAP; green) in the olfactory bulb **(A, B),** cortex **(C, D)** and vestibular nuclei **(E, F)** of *Ndufs4* KO at approximately 60d of age**. G-L)** Reduced astrocyte proliferation in corresponding regions of *Ndufs4* KO mice treated with NV354 (DW, 250 mg/kg/day). Images are representative of n = 3.

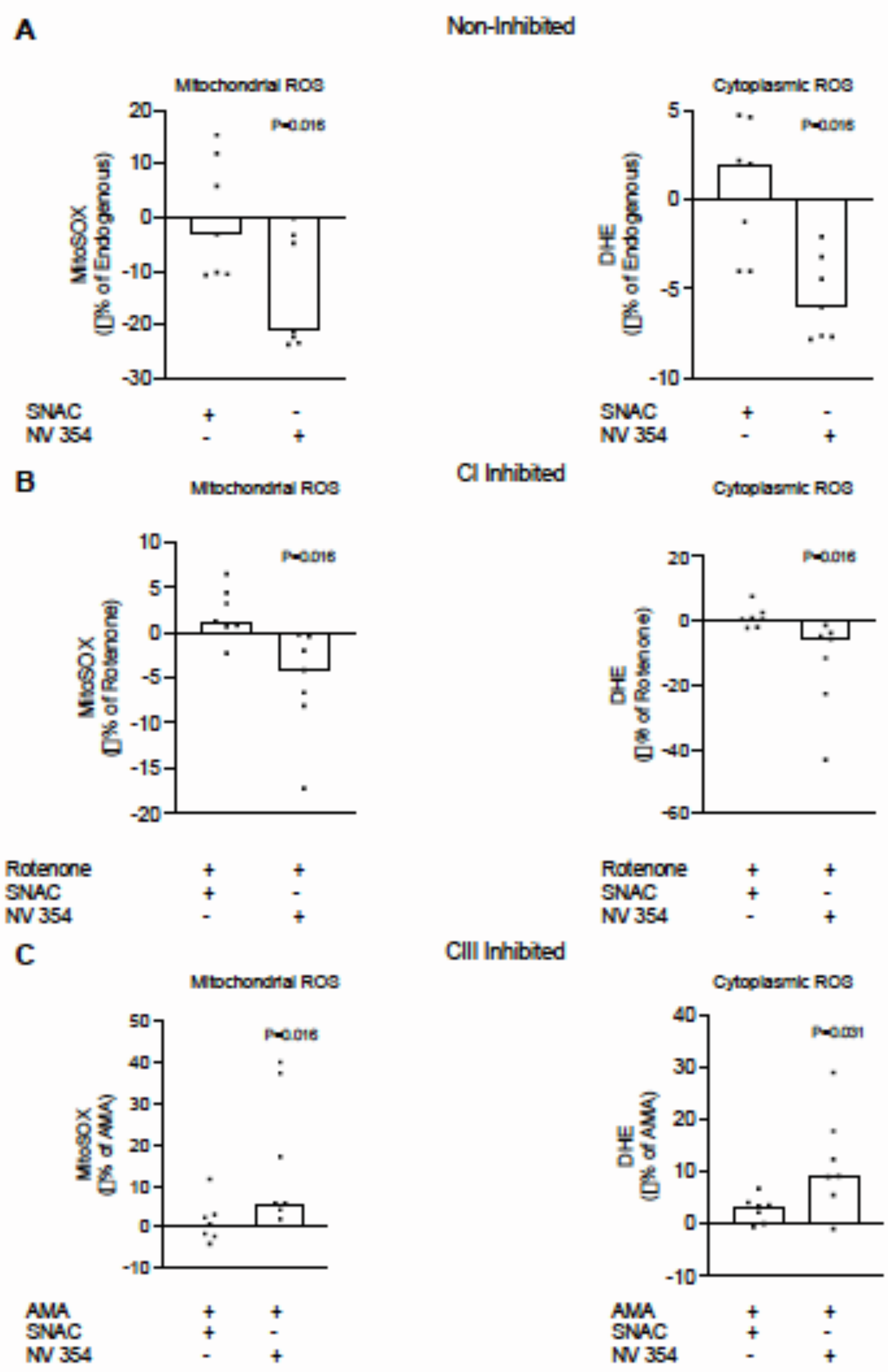
A
Non-Inhibited
Mitochondrial ROS
P=0.016
MitoSOX
(% of Endogenous)
SNAC + -
NV 354 - +
Cytoplasmic ROS
P=0.016
DHE
(% of Endogenous)
SNAC + -
NV 354 - +
B
CI Inhibited
Mitochondrial ROS
P=0.016
MitoSOX
(% of Rotenone)
Rotenone + +
SNAC + -
NV 354 - +
Cytoplasmic ROS
P=0.016
DHE
(% of Rotenone)
Rotenone + +
SNAC + -
NV 354 - +
C
CIII Inhibited
Mitochondrial ROS
P=0.016
MitoSOX
(% of AMA)
AMA + +
SNAC + -
NV 354 - +
Cytoplasmic ROS
P=0.031
DHE
(% of AMA)
AMA + +
SNAC + -
NV 354 - +

Figure S6. NV354 reduces mitochondrial and cytoplasmic reactive oxygen species (ROS) in human primary cells. Flow cytometry of isolated human peripheral blood mononuclear cells (PBMCs) labeled with the mitochondrial-targeted fluorochrome MitoSOX Deep Red (left) and dihydroethidium (DHE) (right). Addition of the succinate prodrug NV354 (100 μM) decreased mitochondrial and cytoplasmic ROS in both non-inhibited (A) and rotenone (2 μM)-treated cells (B), as compared to the cleaved by-product n-acetylcysteamine (SNAC) administered at equimolar concentration. Antimycin A (AMA) treatment (1 mg/mL) provides a complete downstream block of electron transport at complex III. Administration of NV354 increased mitochondrial and cytoplasmic ROS as compared to SNAC. The oxidation level of the fluorochromes is in (A) expressed as Δ% of the endogenous mean fluorescence intensity of vehicle controls, in (B) as Δ% of rotenone-treated samples and in (C) as Δ% of AMA-treated samples. Analyzed by Wilcoxon matched-pairs signed rank test, n=7.

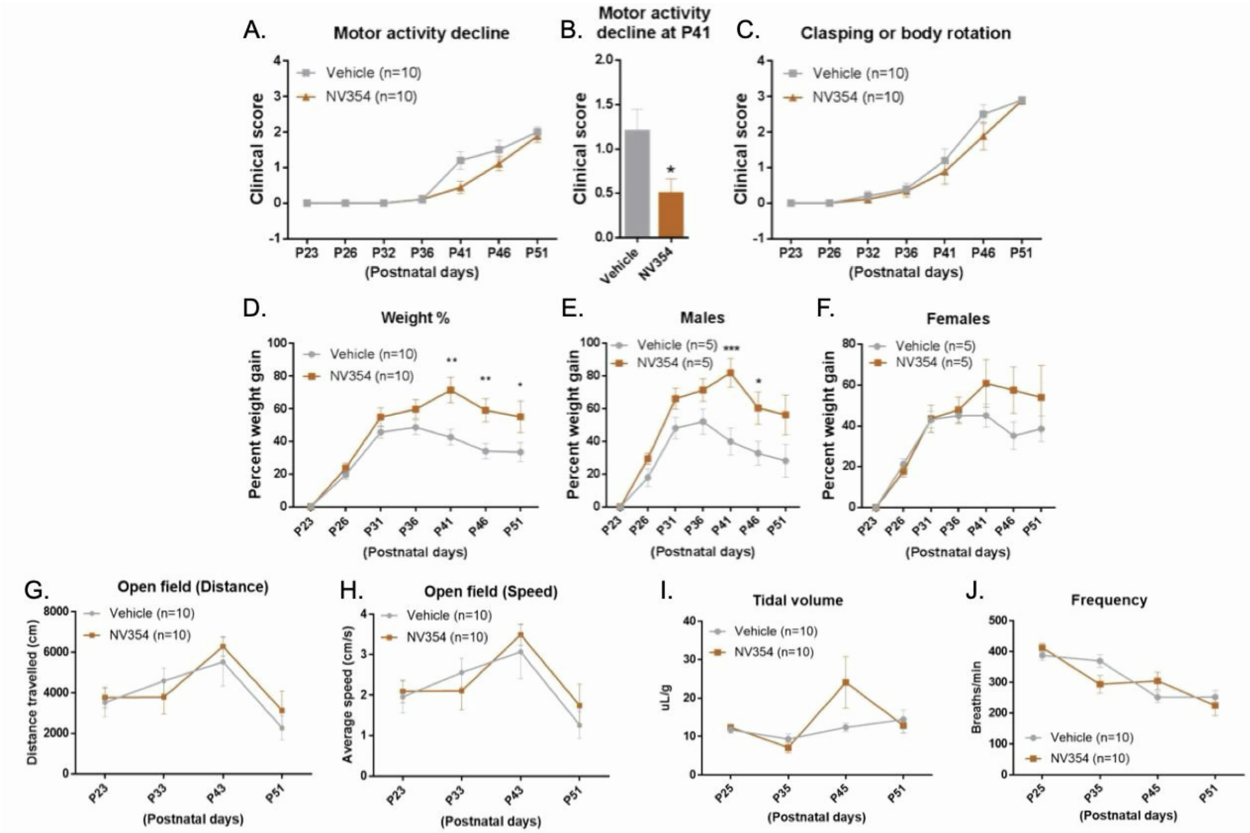

Fig S7. Clinical disease progression in *Ndufs4* KO mice treated with NV354 via osmotic pump (70 mg/kg/day). A) Clinical score representation for motor deficiency onset shows

progressive worsening in vehicle- and NV354-treated *Ndufs4* KO mice during disease progression but (**B**) significant delay of motor decline occurs at postnatal day P41 in NV354-treated mice by unpaired t-test. **C**) Clinical score for manifestation of clasping and/or body rotation reveals no differences when comparing vehicle and NV354-treated *Ndufs4* KO mice. **D**) Percent of body weight gain during disease progression of *Ndufs4* KO mice treated with NV354 is significantly higher than in vehicle group, especially in male mice (**E**) compared to female (**F**). *Ndufs4* KO mice treated with NV354 via osmotic pump do not show statistically significant differences with vehicle group in distance or velocity measured in the open field test (**G-H**) or in respiratory function assessed by tidal volume and breathing rate with whole body plethysmography test (**I-J**). Data is shown in terms of mean ± SEM (*p<0.05, **p<0.01, ***p<0.001) and analyzed by unpaired t-test (**B**) two-way ANOVA followed by Holm-Šídák's posthoc test for multiple comparisons (**A** and **C-F**), n=10.
